# Assessment of Genetic Diversity and Structure of Large Garlic (*Allium sativum*) Germplasm Bank, by Diversity Arrays Technology “Genotyping-by-Sequencing” Platform (DArTseq)

**DOI:** 10.3389/fgene.2017.00098

**Published:** 2017-07-20

**Authors:** Leticia A. Egea, Rosa Mérida-García, Andrzej Kilian, Pilar Hernandez, Gabriel Dorado

**Affiliations:** ^1^Departamento de Bioquímica y Biología Molecular, Campus Rabanales (C6-1-E17), Campus de Excelencia Internacional Agroalimentario (ceiA3), Universidad de Córdoba Córdoba, Spain; ^2^Instituto de Agricultura Sostenible (IAS-CSIC), Campus Alameda del Obispo Córdoba, Spain; ^3^Diversity Arrays Technology Pty. Ltd., Canberra ACT, Australia

**Keywords:** DNA fingerprinting, breeding, phenotype, somatic mutation, second-generation sequencing (SGS), third-generation sequencing (TGS), next-generation sequencing (NGS)

## Abstract

Garlic (*Allium sativum*) is used worldwide in cooking and industry, including pharmacology/medicine and cosmetics, for its interesting properties. Identifying redundancies in germplasm blanks to generate core collections is a major concern, mostly in large stocks, in order to reduce space and maintenance costs. Yet, similar appearance and phenotypic plasticity of garlic varieties hinder their morphological classification. Molecular studies are challenging, due to the large and expected complex genome of this species, with asexual reproduction. Classical molecular markers, like isozymes, RAPD, SSR, or AFLP, are not convenient to generate germplasm core-collections for this species. The recent emergence of high-throughput genotyping-by-sequencing (GBS) approaches, like DArTseq, allow to overcome such limitations to characterize and protect genetic diversity. Therefore, such technology was used in this work to: (i) assess genetic diversity and structure of a large garlic-germplasm bank (417 accessions); (ii) create a core collection; (iii) relate genotype to agronomical features; and (iv) describe a cost-effective method to manage genetic diversity in garlic-germplasm banks. Hierarchical-cluster analysis, principal-coordinates analysis and STRUCTURE showed general consistency, generating three main garlic-groups, mostly determined by variety and geographical origin. In addition, high-resolution genotyping identified 286 unique and 131 redundant accessions, used to select a reduced size germplasm-bank core collection. This demonstrates that DArTseq is a cost-effective method to analyze species with large and expected complex genomes, like garlic. To the best of our knowledge, this is the first report of high-throughput genotyping of a large garlic germplasm. This is particularly interesting for garlic adaptation and improvement, to fight biotic and abiotic stresses, in the current context of climate change and global warming.

## Introduction

Garlic (*Allium sativum*) is a plant producing an edible bulb, made of storage leaves known as cloves. It is of Asian origin, being *Allium longicuspis* considered its wild ancestor. It belongs to genus *Allium*, which includes almost 1,000 species, such as chive (*Allium schoenoprasum*), leek (*Allium ampeloprasum*), onion and shallot (*Allium cepa*) (Maab and Klaas, 1995; Kamenetsky et al., 2004; Meredith, 2008; Cardelle-Cobas et al., 2010; Pacurar and Krejci, 2010). Garlic has a large diploid genome (2*n* = 2*x* = 16), of an estimated haploid (1C) size of 15.9 gigabase pairs (Gbp); that is, 32 times larger than rice (*Oryza sativa*). Garlic is sterile (does not produce fertile botanical seeds by sexual reproduction), asexually propagating by its cloves, despite some progress in recent years to restore garlic fertility (Shemesh-Mayer et al., 2015). Besides, cloves must be reproduced every year, since they cannot be stored for longer periods and then germinated, as happens with standard botanical seeds. Such peculiarity adds extra cost and inconvenience to its maintenance, mainly for large germplasm collections. The peculiar garlic reproduction could lead to low genome diversity, since meiosis is not involved in its clonal reproduction by vegetative propagation (Kamenetsky et al., 2015). Yet, garlic shows a surprisingly high biodiversity, as well as environmental-adaptation capacity and phenotypic plasticity (Volk et al., 2004). All that leads to the large number of garlic varieties or cultivars available (traditionally classified by agromorphological characteristics). The reason for that is not fully understood, suggesting a complex genome (Green, 2001), due to its extremely large size containing many multicopy genes and other duplications, including non-coding sequences and tandem repeats (Arumuganathan and Earle, 1991; Jones et al., 2004; Ovesna et al., 2015), which should be better understood once sequenced. So far, partial and total genome duplications have been described (Supplementary Table S1). Additionally, somatic mutations have been also reported for this species, as well as somaclonal variation, differential gene-expression and alternative splicing (Al-Zahim et al., 1999; Rotem et al., 2007; Kamenetsky et al., 2015; Shemesh-Mayer et al., 2015). Probably, transposable elements are also involved in the evolution of this species.

Besides being appreciated in cooking as common seasoning for thousands of years (Cardelle-Cobas et al., 2010), garlic is also used in pharmacology and cosmetics. Indeed, it is known to have medical properties, protecting against different diseases, like, for instance, hypercholesterolemia, hypertension, atherosclerosis, and thrombosis, reducing the risk of developing cardiovascular disease (CVD). Other recognized bioactivities are antimicrobial (albeit being probiotic), antiasthmatic, antioxidant, anticarcinogenic, etc. (Corzo-Martínez et al., 2007; Pacurar and Krejci, 2010; Rana et al., 2011). Indeed, garlic contains bioactive compounds, including, among others: (i) lectins, which have wide applications in biomedicine and biotechnology (Smeets et al., 1997); (ii) peptides with angiotensin I-converting enzyme (ACE) inhibitory activity, being related to its antihypertensive activity (Suetsuna, 1998); and (iii) *N*-feruloyltyramine, which protects against CVD by suppressing platelet activation (Park, 2009). Besides, this species is rich in enzymes with industrial interest; for instance: (i) nucleases (DNase and RNase), with application in molecular biology (Carlsson and Frick, 1964); (ii) cellulases for biotechnological applications, like conversion of biomass into biofuel (Kim et al., 2010); (iii) superoxide dismutases (SOD), which represent a main defense against oxidative stress, being widely used in pharmacology/medicine, cosmetics, food, agriculture, and chemical industries (He et al., 2008; Liu et al., 2011); (iv) proteases/hemagglutinases, with application in medical tests (Parisi et al., 2008); and (v) alliinases (also known as alliinases), that catalyze conversion of alliin to allicin, which is the main therapeutic agent of garlic (Corzo-Martínez et al., 2007; Kim et al., 2010; Rathnasamy et al., 2014).

On the other hand, agricultural practices usually involve cultivation of a reduced number of species and varieties, which may lead to genetic erosion. That is especially relevant for monocultures, which on the other hand are required to feed an exponentially growing human population. It is therefore important to maintain germplasm banks as reservoirs of genetic variability for crop breeding. Thus, such collections may harbor genetic potential to improve productivity and adaptation/resistance to abiotic (drought, salinity, etc.) and biotic (diseases and plagues) stresses (Tanksley and McCouch, 1997). That is particularly relevant in the current frame of climatic change and global warming. Understanding this potential is critical for identification of biodiversity in biological resources and its efficient management, including conservation and selection of genetically divergent accessions to optimize breeding programs (Olukolu et al., 2012).

Yet, germplasm banks may be generated as mere raw collections of varieties over many years, being classified by criteria based on phenotypic/agronomic traits (passport data). That could lead to both homonymy (same name for genetically different cultivars) and duplications or synonymy (same cultivars with different names). That is especially problematic for species with similar appearance and significant phenotypic plasticity, like garlic. Thus, efficient identification of biodiversity is of paramount importance to manage and maintain such genetic-resources (Govindaraj et al., 2015). That is relevant not only to identify genuine variability for breeding purposes, but also to reduce space and maintenance costs, especially for large germplasm banks, generating reduced, albeit representative, core collections (Zhao et al., 2010).

The role of molecular markers as a tool for genetic analyses and crop improvement has gained importance through the years, as we have reviewed (Dorado et al., 2015c). Their use has become common in model species and important crops. Indeed, genetic diversity and polymorphism assessments are major priorities in plant and crop-breeding studies (Nybom and Bartish, 2000). Large-scale identification of molecular markers like single-nucleotide polymorphism (SNP) on genome and transcriptome represent interesting approaches (Ipek et al., 2016; Akpinar et al., 2017). Classical molecular-markers to assess genetic diversity and polymorphism in garlic have been described (Ovesná et al., 2014; Ipek et al., 2015). Among others, they include isozymes, random-amplified polymorphic DNA (RAPD) (Maab and Klaas, 1995), simple-sequence repeats (SSR) (DaCunha et al., 2014), amplified-fragment length polymorphism (AFLP) (Ipek et al., 2005) and insertions-deletions (InDel) (Wang et al., 2016). Yet, such analyses of genetic diversity in this species are challenging (Kim et al., 2009).

Fortunately, recent technological developments overcome previous limitations. They include second-generation sequencing (SGS) and third-generation sequencing (TGS) approaches, sometimes known by the ambiguous next-generation sequencing (NGS) terminology, as we have reviewed (Dorado et al., 2015b). Thus, a high-throughput genotyping-by-sequencing (GBS) technology (DArTseq) has been developed. It combines diversity arrays technology (DArT) complexity reduction methods with SGS/TGS (Kilian et al., 2012; Courtois et al., 2013; Cruz et al., 2013; Raman et al., 2014), allowing to identify SNP. DArT markers are polymorphic segments of DNA that are found at specific genome sites, after complexity reduction, being detected by hybridization. Those markers may show dominant or codominant inheritance (Gupta et al., 2008). DArT markers exploit DNA-microarray platforms to analyze DNA polymorphisms, without requiring previous DNA-sequence knowledge. Their applications include genetic fingerprinting, like whole-genome profiling for molecular breeding, germplasm characterization and genetic mapping, among others (Jaccoud et al., 2001). DArTseq can be optimized for each organism and application, by selecting the most appropriate complexity-reduction method (both size of representation and fraction of selected genome for assays). This is particularly relevant for garlic, which has a large and expected complex genome, as previously described. Therefore, DArTseq has been used in the present work as a proof-of-concept, to analyze a large garlic-germplasm bank.

The main goals of this study are: (i) assess genetic diversity and structure of a large garlic-germplasm bank; (ii) create a core collection to reduce the number of original accessions, without losing genetic diversity; (iii) relate genotype to agronomical features; and (iv) describe a cost-effective method to manage genetic diversity that could be applied to germplasm banks and breeding projects of garlic and other species.

## Materials and Methods

### Plant Material and DNA Isolation

A total of 417 *a priori* different garlic entries collected in Spain (some of them being originally derived from other countries) were used for DArTseq analyses: 408 from the main Garlic-Germplasm Bank at “Instituto Andaluz de Investigación y Formación Agraria, Pesquera, Alimentaria y de la Producción Ecológica” (IFAPA) of “Junta de Andalucía” in Cordoba; five from Cordoba University (C1 to C5); and four (G, K, L, and M) from “Centro de Ensayos de Evaluación de Variedades” at “Instituto Nacional de Investigación y Tecnología Agraria y Alimentaria” (INIA) in Madrid (Supplementary Table S1). Garlic leaves were frozen in liquid nitrogen and stored at -80°C until needed.

DNA was isolated using cetyl trimethylammonium bromide (CTAB) protocol (Murray and Thompson, 1980), as we have optimized (Hernandez et al., 2001). It was dissolved in Tris-Na_2_EDTA (TE; pH 8) and stored at 4°C. Isolated DNA was quantified by NanoDrop 2000c (Thermo Fisher Scientific, Waltham, MA, United States) and segregated by 1% (w/v) agarose [from United States Biological (Salem, MA, United States)] gel electrophoresis (AGE). Then it was stained with ethidium bromide from Sigma–Aldrich (St. Louis, MO, United States). Resulting DNA was visualized under ultraviolet (UV) light for quality evaluation, using a Molecular Imager VersaDoc MP 4000 System from Bio-Rad (Hercules, CA, United States). Additionally, DNA digestions with the frequent-cutter *Tru1*I restriction enzyme (RE; cutting at T^j^ TA_j_ A) from Thermo Fisher Scientific were performed, in order to check DNA quality and absence of contaminating nucleases.

### DArTseq

DArTseq method from Diversity Arrays Technology (Canberra, ACT, Australia) is described elsewhere^1^. In short, the following steps were carried out: (i) complexity reduction, in which genomic DNA was digested with a combination of restriction enzymes. Then, adapters were ligated and only polymorphic fragments were selected. In this way, this technique allowed to exclusively focus in those sections of the genome which are interesting for genetic-diversity analyses, due to their polymorphism; (ii) polymorphic fragments were cloned into *Escherichia coli* bacteria to create a library. Each *E. coli* colony should contain one of those fragments; (iii) the generated library was amplified by polymerase chain-reaction (PCR), as we have reviewed (Dorado et al., 2015a); (iv) amplicons were cleaned and evaluated by capillary electrophoresis sizing; (v) fragments were sequenced; (vi) A FASTQ file was created with generated sequencing reads, including sequences from 30 to 60 base pairs (bp) of polymorphic fragments; (vii) an internal alignment was performed, using other reads from the library (this step is carried out in case of incomplete or absent reference genome, like in the present work); (viii) SNP and SilicoDArT markers were searched and filtered using algorithms; and (ix) resulting data were two presence/absence (1 and 0, respectively) matrices. One contained SNP and the other SilicoDArT markers, where each column represented an individual and each row a marker (Kilian et al., 2012).

In our case, four methods of complexity reduction were tested in garlic (data not shown), selecting the *Pst*I-*Nsp*I restriction enzymes (cutting at G| TGCA| G and R| CATG| Y, respectively). Briefly, DNA samples were processed in digestion/ligation reactions as previously described (Kilian et al., 2012), but replacing a single *Pst*I-compatible adaptor with two different adaptors, corresponding to two different RE overhangs. The *Pst*I-compatible adapter was designed to include flowcell-attachment sequence from Illumina (San Diego, CA, United States), sequencing-primer sequence and “staggered” barcode (varying-length region), similar to previously reported (Elshire et al., 2011). Reverse adapter contained flowcell-attachment region and *Nsp*I-compatible overhang sequence. Interestingly, an overrepresented sequence from cytoplasmic (chloroplastic) DNA, corresponding to >10% of total sequences, was identified (after initial optimization) in many *Pst*I-*Nsp*I garlic-library samples. A cut site for *Alw*I (cutting at GGATCNNNN| N|) was identified within this overrepresented sequence, and thus such restriction enzyme was included in the digestion-ligation step of library construction. Only “mixed fragments” (*Pst*I-*Nsp*I) which did not have *Alw*I site were effectively amplified in 30 rounds of PCR, using the following reaction profile: (i) denaturation at 94°C for 1 min; (ii) 30 cycles [94°C for 20 s (denaturation), 58°C for 30 s (primer annealing) and 72°C for 45 s (primer extension)]; and (iii) final polymerization at 72°C for 7 min. Equimolar amounts of PCR amplicons from each sample reaction of 96-well microtiter plates were bulked and applied to c-Bot (Illumina) bridge PCR, followed by sequencing on HiSeq 2000 sequencing system from the same manufacturer. Single-read sequencing reactions were run for 77 cycles.

Sequences generated from each lane were processed using DArT analytical-pipelines. In the primary one, Fast-Alignment Sequence Tools Q (FASTQ) files were first processed. Thus, poor-quality sequences were filtered-away, applying more stringent selection criteria to the barcode region, as compared to the rest of the sequence. Assignments of sequences to specific samples in the “barcode split” step were very reliable. This way, approximately 2,000,000 sequences per barcode/sample were identified and used in marker calling. Finally, identical sequences were collapsed into “fastqcoll” files. These were “groomed” using the DArT PL’s C++ algorithm, which corrects low-quality bases from singleton-tags into correct bases, using collapsed tags with multiple members as template.

Groomed fastqcoll files were used in the secondary pipeline (presence/absence of restriction fragments in representation), by DArT, PL, SNP, and SilicoDArT calling algorithms (DArTsoft version 14). In total, 33,423 presence/absence markers were generated. All tags from all libraries included in the DArTsoft analyses were clustered using the DArT PL’s C++ algorithm (threshold distance of 3), for SNP calling. That was followed by cluster parsing into separate SNP loci, using a range of technical parameters; especially the balance of read counts for allelic pairs. Additional selection criteria were added to the algorithm, based on previous experience with analyses of approximately 1,000-controlled cross populations (data not shown). Testing for Mendelian distribution of alleles in these previous populations facilitated selection of technical parameters, discriminating well-true allelic variants from paralogous sequences. In addition, multiple samples were processed from DNA to allelic calls, as technical replicates and scoring consistency was used as the main selection criteria for high-quality/low error-rate markers. Calling quality was assured by high average-read-depth per locus (average across all markers was over 10 reads/locus).

### Genetic Diversity and Structure Assessments

Three different analyses were performed, in order to study genetic diversity and structure of germplasm-bank accessions. After creating the SNP and SilicoDArT marker scoring matrices, a Gower’s distance matrix was generated. Gower’s distance is a coefficient that measures similarity between two samples, based on logical (absence/presence) information differing for several variables (Gower, 1971). These data were used to determine genetically redundant samples. Secondly, a hierarchical cluster-analysis was done with the “pvclust” R package (Suzuki and Shimodaira, 2015). The phylogenetic tree (dendrogram) was computed with a complete-linkage method. By doing complete-linkage clustering (agglomerative hierarchical clustering method), each element of a distance matrix was first individually clustered. Then, each sample was combined into a new cluster, according to the shortest distance (Defays, 1977). Besides previous tests, a principal-coordinates analysis (PCoA; also known as classical multidimensional scaling, Torgerson Scaling or Torgerson-Gower scaling) was also carried out, using R software version 3.2.2 (R-Development-Core-Team, 2015). Additionally, STRUCTURE software version 2.3.4 (Pritchard et al., 2000) was used to study genetic structure. The chosen parameters were five iterations, *K* ranging from 1 to 3, with a burnin length of 10,000 and 20,000 Markov Chain Monte Carlo (MCMC) repetitions after burnin.

## Results

### DArTseq Analyses

A total of 417 garlic samples were analyzed using SilicoDArT markers (representing presence/absence of restriction fragments in DArT genomic representations) and SNP data. A total of 14,392 SNP were used for the analyses. DArTseq markers allowed identifying 286 unique (Supplementary Table S2) and 131 redundant samples. The latter were divided into 19 groups, showing a variable amount of individuals (two to 53; Supplementary Table S3). For instance, in group 1, samples 717 and 718 were from the same province (Jaen, Spain). Spanish White varieties were mainly associated in groups 2 and 3 (samples 238, 452, and 461, all from northern Spain). Additionally, for group 2, there was an internal structure between regions. Samples 335, 424, 433, 434, 457, 464, and 467 were from northern Spanish provinces; samples 360 and 368 came from Caceres (Spain) and samples 127, 130, and 553 from southern Spanish provinces. Groups 4 and 7 to 10 included Spanish Purple varieties. Particularly, samples in group 4 were all from Castilla-Leon (Spain). Group 7 was the most numerous, with a total amount of 53 redundant samples. Interestingly, some associations by province were found in this group. Thus, samples 2, 59, 486, and 489 were all from northern regions; samples 21, 37, and 366 from central provinces; and samples 3, 85, 107, 110, 125, 131, 139, 150, 171, 225, 344, 356, 715, and 720 were from southern provinces. Two samples (14 and 280) from Taiwan, were also included in group 7. On the other hand, no associations were found for groups 5, 6, and 11 to 19.

### Germplasm-Diversity Assessments

The 417 garlic samples were further analyzed, in order to assess their genetic diversity and structure, to eliminate redundant accessions, and thus generate the germplasm-bank core collection. Two different analyses were performed: hierarchical cluster computed by complete-linkage method and PCoA. The dendrogram (**Supplementary Figure S1**) showed three main clusters (I to III), besides a few samples diverging from them (A and B). Main branches were supported by high-bootstrap values (>90). Moreover, bootstrap values were mainly high as well inside the main three clusters. Only some final subgroups had statistically non-significant bootstrap values. The separation in the dendrogram of some well-characterized samples (C1 to C5) is of special interest. Thus, Spanish varieties (Purple C3 and White C4; highlighted in purple and pink, respectively, in **Supplementary Figure S1**) were more related between them than to Chinese varieties (White C1 and Purple C2; highlighted in brown in **Supplementary Figure S1**), which were closely related. Sample C5 is a Brazilian garlic (thought to be an old Spanish Purple variety exported to America during colonialism) brought back to Spain 5 years ago. Interestingly, it was nearer to Spanish samples (closer to C3 than to C4) than to other accessions (C1 and C2), being highlighted in purple (**Supplementary Figure S1**).

Agro-morphological information (Supplementary Table S1) showed data in agreement with the generated dendrogram. For instance, cluster A contained samples 167, 239, and 459, being hexaploid or giant varieties (**Supplementary Figure S1**; highlighted with orange dots). There was a fourth hexaploid individual (379), being located in cluster III. Another interesting case was made of samples grouped together and with similar geographical origins. Thus, accessions 511, 513, and 514 came from Egypt (**Supplementary Figure S1**; highlighted with brown dots). Additionally, there were clusters with samples from Castilla-Leon region like: (i) 380, 389, and 432; (ii) 376, 424, 425, and 431; and (iii) 54, 423, 434, and 438 in the case of cluster II (highlighted with pink dots). Samples 32, 123, 125, 136, 225, and 1390 in cluster III were from Andalusia region (Spain; highlighted with purple dots). Samples 265, 270, 272 to 274, 276, 300, and 373 from cluster B came from Japan.

In addition, most accessions were also grouped by garlic-variety color in the phylogenetic tree. Thus, samples 20, 54, 238, 335, 360, 368, 424, 452, and 467 were Spanish White varieties (cluster II, pink). Likewise, samples 2, 3, 16, 17, 19, 21, 27, 29, 30, 32, 33, 37, 38, 77, 85, 87, 110, 117, 120, 123 to 125, 131, 132, 136, 138 to 141, 149, 150, 158, 161, 166, 171 to 173, 296, 297, 342, 343, 349, 356, 366, 454, 489, 542, 543, 560, 566, 570, 572, 574, 577, 578, 694, 752, 774, 779, G and K were Spanish Purple, Red, Brown, or “Colorado” varieties (cluster III, purple). Conversely, some samples did not group as expected. Thus, accessions 176 and 353 (Brown and Spanish Purple, respectively) would belong to cluster III, in accordance to their available agro-morphological data, yet they were in cluster A. Likewise, samples 36, 43, 88, and 109 (being considered Red or Purple varieties) did not group in cluster III, but in cluster II instead. Additionally, sample 44 is described as Chinese and thus expected in cluster I, but showed in cluster II instead. Samples 28, 79, 101, 137, 268, 526, 753, 776, and L (described as White varieties) were expected in cluster II, but were in cluster III. Sample 51 (described as Spanish White) was conversely located in cluster I instead of II. Likewise for some Spanish Purple samples (7, 348, 363, 369, and 775). Finally, samples 263 and 300 (described as White varieties) were included in cluster B instead of II. All samples that were not assigned consistently with agro-morphological data were highlighted with red dots in **Supplementary Figure S1**.

**FIGURE 1:**
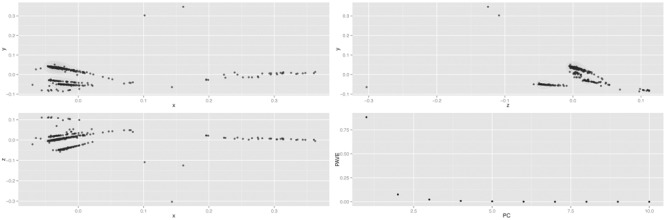
Garlic principal-component plot. PCoA analysis was carried out to further analyze the garlic germplasm diversity. Upper-left represents principal coordinate (PC1; *x*-axis) with PC2 (*y*-axis); lower-left compares PC1 (*x*-axis) to PC3 (*z*-axis); and upper-right shows PC2 (*z*-axis) versus PC3 (*y*-axis). The lower-right graph corresponds to the Proportion of Analysis of Variance Explained (PAVE).

Principal-coordinates analysis was performed to further evaluate dendrogram clusters (**Figure 1**). Variance (genetic diversity) explained by principal components (PC) (accounting for 0.99 of cumulative variance) was 0.93 for PC1, 0.04 for PC2, and 0.02 for PC3. The relationships for samples C1 to C5 were similar to the ones in the dendrogram. As expected, samples C1 and C2 were nearer among them (Chinese), as well as samples C3 to C5 (Spanish origin). In addition, samples C3 and C5 were also closer compared to C4, as displayed in dendrogram (Supplementary Table S4).

### Germplasm Genetic-Structure

Genetic structure of the garlic germplasm-bank collection was evaluated with STRUCTURE software. Three groups were found, based on maximum likelihood and delta *K* (Δ*K*) values (**Supplementary Figure S2a**). As described above, this result is in agreement with cluster analysis and PCoA. Bar plot for *K* = 3 was also shown (**Supplementary Figure S2b**). In relation to the probability of membership of samples to clusters, Cluster I showed a score of 44.8%, being the group with the highest percentage. Clusters 2 and 3 had similar values (26.4 and 28.8%, respectively). When the probability of belonging to a group was high (≤0.8 to 0.9), such individuals showed the same association found in hierarchical cluster-analysis. Well-known varieties (C1 to C5), also maintained the same relationships (Supplementary Table S5).

## Discussion

Garlic is known for multiple alimentary, medical and cosmetic uses worldwide. Yet, its classification and conservation in germplasm banks is challenging, due to homonymy and synonymy, being further complicated by its asexual life-cycle (Ipek et al., 2005). Previous information available allowed classifying the studied germplasm samples in this work by agro-morphological traits. Yet, such approach may be non-effective identifying true biodiversity, increasing redundancies and thus space and preservation costs in germplasm banks. In fact, it is known that the same garlic genotypes in different environmental conditions could exhibit diverse phenotypes (Volk et al., 2004). This is due to the high phenotypic plasticity of garlic, probably linked to its huge and expected complex genome, which somehow should compensate its lack of sexual reproduction.

Molecular markers have become an essential tool to identify, manage, and protect genetic diversity. Yet, developing them may be complicated, time-consuming and expensive for species like garlic, without sequenced reference genome, in which only scarce genomic-information is available (Ovesná et al., 2014). Additionally, classical molecular markers like isozymes, RAPD, SSR, or AFLP are not well suited to genotype garlic germplasm banks, due to its lack of resolution for such a peculiar genome in asexually reproducing accessions. Fortunately, technologies like DArT –and more recently, DArTseq– allow to reduce complexity and thus resolve complex genomic samples (Jaccoud et al., 2001).

Therefore, DArTseq was used in the present work to evaluate the genetic diversity and structure of 417 garlic samples (408 accessions from a garlic-germplasm bank). Data were analyzed by hierarchical-cluster computed by complete-linkage method, PCoA and genetic-structure approaches. Results showed a general consistency between accessions, geographic origins and groupings for expected/known garlic identities. All tests showed that individuals could be divided into three main groups (I, II, and III). Moreover, when the statistical probability of belonging to a group was high, the same association pattern of individuals was found in hierarchical-cluster analysis. Specifically, patterns for samples C1 to C5 (according to the previously known information) were maintained. Hence, DArTseq markers proved to be an effective and consistent genotyping approach to assess genetic diversity and structure.

Samples grouped by variety or geographical proximity were also found in non-redundant accessions, as described in the “Results” section. As expected, garlic samples of the same or near geographical regions grouped together. Indeed, cultivated varieties are usually selected by growers for several reasons, including being adapted to the climate in a specific region. In addition, the asexual garlic reproduction could lead to less genetic diversity and differentiation among varieties with similar geographical origins or different variants of the same variety. On the other hand, some samples were not grouped as expected, according to their agro-morphological information. Yet, such data is generated *de visu*, being therefore less accurate than molecular studies. In fact, it is known that morphological data are not always reliable to classify and detect genetic variation in germplasm collections (Jansky et al., 2015).

On the other hand, STRUCTURE assumes that markers are not in linkage disequilibrium (LD) within subpopulations. Yet, there are redundant lines in the data set, which could be against such assumption. But, there was a high consistency when comparing dendrogram clusters with those generated by STRUCTURE software. Thus, individuals assigned to the same cluster in the former, usually had higher probabilities to belong to the same group in the latter. Only three individuals were assigned differently in such analyses (4, 43, and 430) (Supplementary Table S5 and **Supplementary Figure S2**). This could be due to several reasons. In fact, criteria and calculations could lead to different results in each analysis. In the case of samples 4 and 430, they were located in an initial branch of cluster III, which indicates that they were genetically more different that the rest of assigned samples. Additionally, agro-morphological information was missing for samples 4 and 430.

The redundancy analysis showed that about one third of studied samples (131) could be considered as genetically redundant vs. 286 non-redundant (unique). This shows the higher resolution power and value of genomic analyses over agro-morphological ones. Thus, DArTseq results allowed to significantly reduce the analyzed garlic germplasm-bank size by 31.41%, generating a core collection, which was the main purpose of this research. Redundant accessions were divided into 19 groups (Supplementary Table S3). Samples included in each of them were in general related by variety (White, Purple, etc.) or location (same or near provinces). Interestingly, White varieties were more differentiated by location, whereas Purple ones were mainly associated in only one group. Samples 79 (Chinese White variety) and 526 (Spanish White variety) showed in group 7, in which Spanish Purple individuals were included. Curiously, this same lack of correlation was found in the hierarchical-cluster analysis, suggesting identities/differences not yet well understood. Further research is required to properly assess such results, including analyses of full genome sequences, once available in the future. That is now a possibility for large genomes like the garlic one, thanks to the throughput increase and cost reduction of TGS, which is expected to become a mature technology in the next years (Dorado et al., 2015b).

As we have found, DArTseq is a cost-effective genotyping tool for creating and maintaining germplasm banks, allowing to properly ascertain, manage and maintain available biodiversity. Such technology has generated high-quality whole-genome profiles and genetic patterns, with dramatically increased resolution in relation to previous methodologies. Additionally, the high number of samples analyzed in this work, together with the large amount of marker data generated on lines with phenotypic information, should be useful for both genetic dissection of important traits and to help breeders improve this crop. Moreover, results obtained by DArTseq in any species can help to perform further analyses in germplasm collections without previous genetic information, even with high phenotypic-plasticity, complex genomes and asexual reproductive-systems that may hamper diversity analyses (Gebhardt, 2013). DArTseq sequences can be used to develop DArTseq markers and other molecular markers, such as SSR or SNP, which can be transferable to other germplasm banks (Belaj et al., 2011; Atienza et al., 2013). These tools can be associated to traits of interest, and thus used for marker-assisted breeding.

## Conclusion

We have significantly reduced the analyzed garlic germplasm-bank size, identifying redundant accessions and thus generating a unique (non-redundant) core collection, with the consequent reduction in space and maintenance expenses. To our knowledge, this is the first work of high-throughput garlic genotyping. The obtained results show that DArTseq is a cost-effective method to perform genotyping-by-sequencing and genetic diversity analyses of such species with huge, expected complex and mostly unknown (without reference) genome, with clear applications for biodiversity conservation. This supports previous studies for characterizing and managing germplasm banks of other species. DArTseq has generated consistent results, in accordance with variety and geographical origin. They remark the relevance of genetic versus agro-morphological data, especially in the context of peculiar garlic-plasticity for environmental adaptation. Additionally, the high number of samples analyzed in this work and the amount of data generated should be useful for plant breeders in general, as well as for garlic adaptation and improvement in particular. This, along with other molecular markers and agro-morphological information represent useful tools to improve management strategies in germplasm-banks. In fact, having a core collection of characterized genotypes and phenotypes could help breeders to select plants with better adaptability. This is important for productivity and to face biotic and abiotic stresses, to fight the current climate change and global warming.

## Author Contributions

LE performed experiments, analyzed data, and wrote the manuscript; RM-G analyzed data and participated in manuscript writing; AK contributed to reagents, analysis tools, and manuscript writing; PH contributed to experimental design, materials, reagents, analysis tools, and manuscript writing; GD conceived and designed the experiments, contributed to materials, reagents, analysis tools, and manuscript writing; All authors read and approved the final version of the manuscript.

## Conflict of Interest Statement

AK works at Diversity Arrays Technology. This fact did not interfere with the objective, transparent and unbiased presentation of results, and does not alter the authors’ adherence to all theoretical and applied genetics policies on data and material release. The other authors declare that the research was conducted in the absence of any commercial or financial relationships that could be construed as a potential conflict of interest.

## Footnotes

1 http://www.diversityarrays.com/dart-application-dartseq

## Supplementary Material

FIGURE S1 Garlic dendrogram. Phylogenetic tree, with approximately unbiased (AU; red)/Bootstrap Probability (BP; green) percentage values and Euclidean distances, generated by complete-linkage method, to ascertain germplasm diversity. Cluster I includes C1 and C2 (Chinese varieties); Cluster II has C4 (Spanish White variety); and Cluster III shows C3 to C5 (Spanish Purple and Brazilian varieties). Samples C1 to C5, and others described in the text, are highlighted with colored dots. I corresponds to cluster II in STRUCTURE analysis, whereas II and III are equivalent to cluster I; and A and B correspond to cluster III using such software analysis.

FIGURE S2 Garlic genetic structure. STRUCTURE software was used to analyze the studied garlic germplasm. **(a)** Diagram showing the three calculated clusters (*K* = 3); and **(b)** Δ*K* values.
