# Supplementary figures and images for: Assessment of Genetic Diversity and Structure of Large Garlic (Allium sativum) Germplasm Bank, by Diversity Arrays Technology “Genotyping-by-Sequencing” Platform (DArTseq)
Source: Front Genet. 2017 Jul 20;8:98. doi: 10.3389/fgene.2017.00098 (PMC5517412; doi:10.3389/fgene.2017.00098)

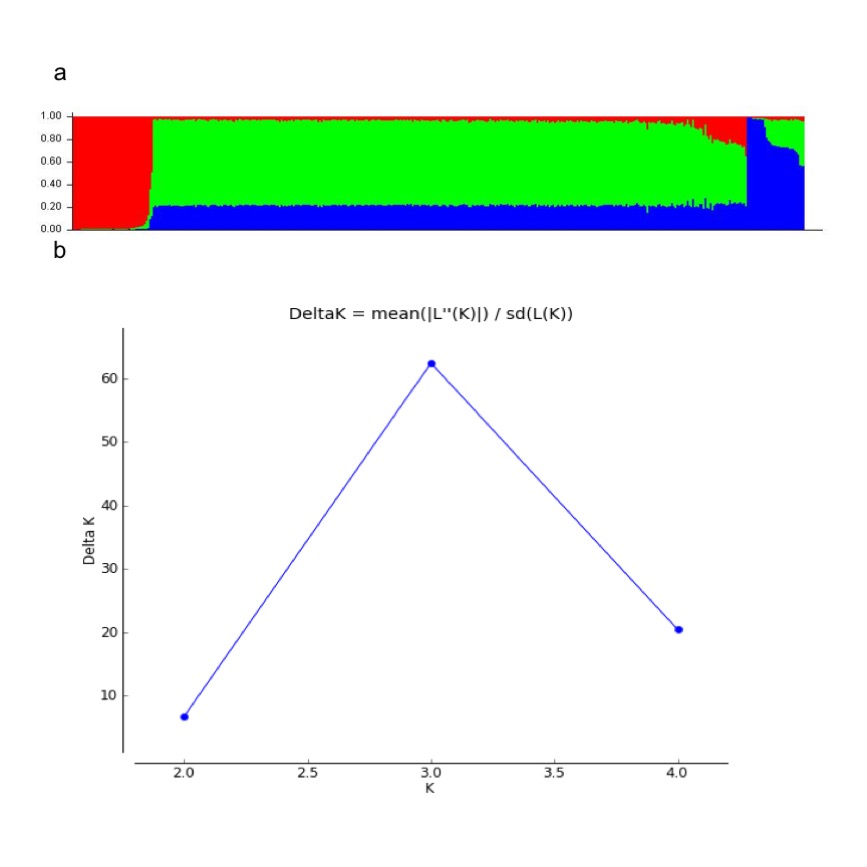

Supplement: FIGURE S2 — Garlic genetic structure. STRUCTURE software was used to analyze the studied garlic germplasm. (a) Diagram showing the three calculated clusters (K = 3); and (b) ΔK values. [file Image_2.jpeg]
